# First Identification and Description of Rickettsioses and Q Fever as Causes of Acute Febrile Illness in Nicaragua

**DOI:** 10.1371/journal.pntd.0005185

**Published:** 2016-12-30

**Authors:** Megan E. Reller, Ijeuru Chikeka, Jeremy J. Miles, J. Stephen Dumler, Christopher W. Woods, Orlando Mayorga, Armando J. Matute

**Affiliations:** 1 Division of Infectious Diseases, Department of Medicine, Johns Hopkins University School of Medicine, Baltimore, Maryland, United States of America; 2 Hubert-Yeargan Center for Global Health, Durham, North Carolina, United States of America; 3 Department of Pathology, University of Maryland School of Medicine, Baltimore, Maryland, United States of America; 4 Duke University School of Medicine, Durham, North Carolina, United States of America; 5 Division of Infectious Diseases and International Health, Department of Medicine, Duke University School of Medicine, Durham, North Carolina, United States of America; 6 Hospital Escuela Oscar Danilo Rosales Arguello, Universidad Nacional Autonoma de Nicaragua, León, Nicaragua; Fondation Raoul Follereau, FRANCE

## Abstract

**Background:**

Rickettsial infections and Q fever present similarly to other acute febrile illnesses, but are infrequently diagnosed because of limited diagnostic tools. Despite sporadic reports, rickettsial infections and Q fever have not been prospectively studied in Central America.

**Methodology/Principal Findings:**

We enrolled consecutive patients presenting with undifferentiated fever in western Nicaragua and collected epidemiologic and clinical data and acute and convalescent sera. We used ELISA for screening and paired sera to confirm acute (≥4-fold rise in titer) spotted fever and typhus group rickettsial infections and Q fever as well as past (stable titer) infections. Characteristics associated with both acute and past infection were assessed.

**Conclusions/Significance:**

We enrolled 825 patients and identified acute rickettsial infections and acute Q fever in 0.9% and 1.3%, respectively. Clinical features were non-specific and neither rickettsial infections nor Q fever were considered or treated. Further study is warranted to define the burden of these infections in Central America.

## Author Summary

Rickettsial infections and Q fever cause illness characterized by fever and non-specific symptoms and signs. Not only are these infections difficult to recognize, they are also difficult to diagnose because of limitations in existing tests for them. Despite sporadic reports, rickettsial infections and Q fever have not been prospectively studied in Central America. We enrolled consecutive patients presenting with undifferentiated fever in western Nicaragua and collected data regarding potential risk factors as well as symptoms and signs associated with the illnesses. Additionally, we collected blood samples at the initial visit and 2 to 4 weeks thereafter. We used serologic assays to differentiate new (rising antibody titers) vs. old (stable antibody titers) infections. Characteristics associated with both acute and past infection were assessed. We enrolled 825 patients and identified acute (new) rickettsial infections and acute Q fever in 0.9% and 1.3%, respectively. Clinical features were non-specific and neither rickettsial infections nor Q fever were considered nor treated. Further study is warranted to define the burden of these infections in Central America.

## Introduction

Rickettsioses, including spotted fever group (SFGR) and typhus group (TGR), and Q fever (caused by *Coxiella burnetii*) are increasingly recognized worldwide [1]. Both rickettsioses and Q fever often manifest as undifferentiated fever and cannot easily be distinguished clinically from other causes of acute febrile illness (AFI). Furthermore, both are difficult to confirm in the laboratory, since convalescent sera, specific diagnostic reagents, and expertise are required. These infections are especially underappreciated in low resource settings where lack of laboratory capacity limits both individual diagnosis and validation of clinical acumen. Under-recognition may lead to unnecessary morbidity and even mortality, since empirical regimens for AFI usually do not treat rickettsioses or Q fever.

In 1971 a large serosurvey documented the presence of rickettsiae and Q fever in humans in Central America [2]. However, these agents have not been prospectively studied in humans in Central America nor have there been cases reported of acute infection with these agents in Nicaragua.

To identify, quantify, and characterize potentially treatable rickettsioses and Q fever among AFI in Nicaragua, we studied a cohort of children and adults presenting with fever at a large hospital.

## Methods

### Ethics statement

Written informed consent was obtained from patients or their guardians (for patients <18 years of age) and written assent was obtained from patients aged 12–17 years. The institutional review boards of Johns Hopkins University and Duke University Medical Center (USA) as well as Universidad Nacional Autónoma de Nicaragua, León (Nicaragua) approved the study.

### Setting and patients

We recruited patients in the emergency department and adult and pediatric wards of Hospital Escuela Oscar Danilo Rosales Arguello (HEODRA), the 400-bed primary public teaching hospital of Universidad Nacional Autónoma de Nicaragua in León, Nicaragua, which serves rural areas around León as well as the city itself. Between August 2008 and May 2009, we enrolled consecutive febrile (≥38°C, tympanic) patients ≥1 month old without prior (within 1 week) trauma or hospitalization who presented during the day or early evening hours Monday through Saturday. Dedicated study doctors verified eligibility and willingness to return for follow-up and obtained written informed consent from patients (≥18 years) or parents (<18 years), and assent if 12–17 years. Study personnel recorded structured epidemiological and clinical data, including the duration of illness and clinical provider’s presumptive (leading clinical) diagnosis, on a standardized form at enrollment and then obtained peripheral blood specimens in EDTA and in a serum-separator tube for on-site clinician-requested testing and off-site research-related testing. Patients returned for clinical and serologic follow-up 2 to 4 weeks later, or were visited at home if they did not return and could be located. Blood in the serum separator tube was centrifuged and sera and EDTA blood frozen on site at -80°C.

### Laboratory testing

#### Samples

Serum and EDTA-anti-coagulated blood samples were stored promptly at -80°C. Samples were shipped on dry ice to and within the United States to diagnose rickettsial and Q fever infections.

#### Serological screening by ELISA for rickettsial infection

Convalescent sera were tested for the presence of IgG antibodies to *Rickettsia rickettsii* using PanBio Spotted Fever Group IgG ELISA (PanBio, Brisbane, Australia) [3] and *Rickettsia typhi* by ELISA [4] using whole cell antigen. Optical density (OD) measurements were adjusted for plate-to-plate variation with a correction factor obtained from ratios of identical positive and negative controls used on each plate.

#### Serological screening by ELISA for Q fever

Convalescent sera were tested for the presence of specific Phase 2 IgG antibodies by ELISA (Institut Viron Serion GmgH, Warburg, Germany) after removal of rheumatoid factor per the manufacturer’s instructions. The assay provided semi-qualitative results—positive, negative, and equivocal. Using a standard curve and evaluation table provided with the kit, optical density (OD) measurements were adjusted for plate-to-plate variation with a correction factor as per manufacturer’s procedure [5].

#### Indirect fluorescent antibody (IFA) to confirm rickettsial infections and Q fever

We tested *R*. *rickettsii* and/or *R*. *typhi* ELISA-positive convalescent sera for antibodies to both SFGR and TGR by standard indirect immunofluorescence (IFA) with *R*. *rickettsii* and *R*. *typhi* antigens (each well with 2 individual antigen spots, slides from Focus Diagnostics, Cypress, California) in addition to *R*. *parkeri* whole cell antigen (courtesy C. Paddock, CDC) prepared in-house (see below). We similarly tested Q fever ELISA-positive convalescent specimens by IFA, using cell-free *C*. *burnetii* phase 2 antigen (courtesy D. Raoult, Marseille, France) fixed to multiwell Teflon-coated glass slides. For all IFA test, patient sera and positive and negative controls were diluted 1:80 with PBS and 0.5% non-fat dry milk, incubated for 30 min at 22˚C with antigens, washed 3 times with PBS, and then incubated with FITC-labeled anti-human IgG (γ heavy-chain specific) for 30 min at 22˚C. Slides were rinsed again 3 times in PBS and counter-stained with Evans blue, dried and mounted with Vectashield H-1000 mounting medium (Vector Laboratories, Burlingame, CA) before examination by epifluorescence. Convalescent sera reactive at the 1:80 screening dilution were titered to end point using serial two-fold dilutions. Corresponding acute-phase sera for convalescent samples with titers ≥80 were screened and titered by IFA as for convalescent samples. Some samples were also tested for antibodies to *R*. *parkeri* Portsmouth strain (courtesy C. Paddock, CDC) by IFA, using *R*. *parkeri* propagated in human brain microvascular endothelial cells affixed to 10 well Teflon coated glass slides by acetone fixation. The IFA procedure was identical to that for *R*. *rickettsii*.

### Case definitions

We used stringent criteria to define a confirmed case [6].

Confirmed acute rickettsial infection. ≥4 fold rise in IgG titer for SFGR and/or TGR;

Confirmed past rickettsial infection. IgG titer by IFA of ≥160 for SFGR and/or TGR in the absence of a ≥4-fold rise in titer.

Confirmed acute Q fever. ≥4 fold rise in *C*. *burnetii* Phase 2 IgG titer by IFA.

Confirmed past Q fever. *C*. *burnetii* Phase 2 IgG titer of ≥160 by IFA in the absence of a ≥ 4-fold rise in titer.

Probable acute rickettsial infection or Q fever. Seroconversion without a ≥4 fold rise in titer (e.g., acute-phase sera negative at 1:80 and convalescent sample positive at 1:80.

Probable past rickettsial infection or Q fever. IgG titer of 80 in the absence of seroconversion or 4-fold rise in titer. Those with equal SFGR and TGR titers were SFGR/TGR group-indeterminate.

Possible acute rickettsial infection or Q fever. 2-fold rise in titer (e.g. acute sample positive at 1:80 and convalescent sample positive at 1:160)

SFGR vs. TGR infection. A ≥2-fold difference in titer defined SFGR vs. TGR; if titers were equal, the rickettsial infection was categorized as group indeterminate

### PCR to confirm acute rickettsial and Q fever infections

Acute phase EDTA-anticoagulated blood samples from patients with possible, probable, and confirmed acute rickettsial and/or Q fever infections (IFA-confirmed seroconversion with convalescent titer ≥ 80 and/or a ≥2-fold rise in titer) as well as past infections were used to prepare DNA for PCR analyses. For this, 1 mL of EDTA-anticoagulated blood was subjected to automated DNA preparation using a QIAsymphony SP instrument and the DSP DNA mini Kit; the final elution volume was 200 μL. DNA samples were initially tested using a multiplex 5’ nuclease assay targeting conserved regions in SFGR *ompA*, TGR 17 kDa genus-common antigen gene (RURANT17KB), and human *ACTB* [7]. In addition, acute phase blood DNA samples from patients with a convalescent-phase SFGR and/or TGR IFA titer of ≥ 160 and a ≥ 2 fold increase in titer were tested separately for SFGR hypothetical protein gene RC0338 [8], TGR hypothetical protein gene *Rpr 274P* [9], and *C*. *burnetii* IS1111 spacer PCR (courtesy D. Raoult, Unite des Rickettsies, Marseille, France) [10].

### Clinical correlation and statistical analysis

We correlated epidemiologic and clinical findings with serologic results. Proportions were compared by the Chi-square test or Fisher’s exact test and continuous variables by Student’s t-test or analysis of variance if normally distributed and Wilcoxon-Mann-Whitney or Kruskal-Wallis test if not normally distributed. Analyses were done with Stata IC 11.0 (StataCorp, College Station, TX).

## Results

### Patient characteristics

Serologic testing for rickettsial infections and Q fever was completed for 800 (97.0%) of 825 consecutively enrolled patients. Of these 800, 748 (90.7%) had paired sera available, since 52 patients did not return and could not be located for follow-up. The likelihood of a subject returning for convalescent serum sampling and clinical follow-up did not differ by age (p = 0.90), sex (p = 0.93), or self-reported urban vs. rural residence (p = 0.53). The reported median distance from residence to hospital was 2 km for those who followed up versus 3 km for those who did not (p = 0.08). Among the 748 patients with paired sera, the median age was 9 years (IQR 3–29). Slightly more were male (52.5%), and males were younger than females (median age 9 vs. 11, p = 0.007). The median reported duration of fever and of illness at presentation was 2 days (IQR 1–4) and 3 days (IQR 1–5), respectively. Many (30.0%) reported taking an antibiotic before presentation. The median interval between acute and convalescent follow-up was 15 days (IQR 14–28). A total of 5 patients were treated with doxycycline, none of whom had acute rickettsial infection or acute Q fever.

### Laboratory testing

#### Serological screening by ELISA for rickettsial infections (Figs 1 and 2)

**Fig 1:**
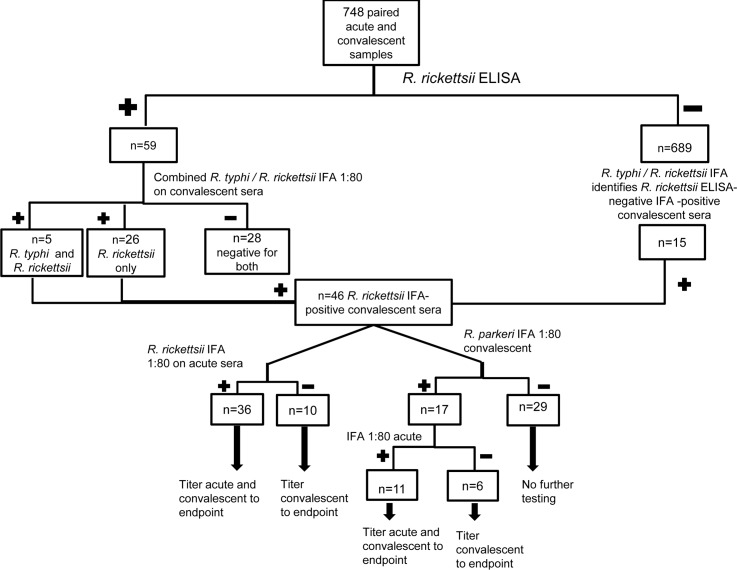
Flow diagram depicting approach to serological evaluation of convalescent and acute phase sera for IgG antibodies to spotted fever group rickettsiae, including ELISA and IFA for *R*. *rickettsii* and IFA for *R*. *parkeri*. Note that the IFA substrate slides used resulted in simultaneous tests for both *R*. *rickettsii* and *R*. *typhi*; those samples identified with *R*. *typhi* IFA antibodies were then included in the *R*. *typhi* tally (Fig 2) if not *R*. *typhi* ELISA reactive.

**Fig 2:**
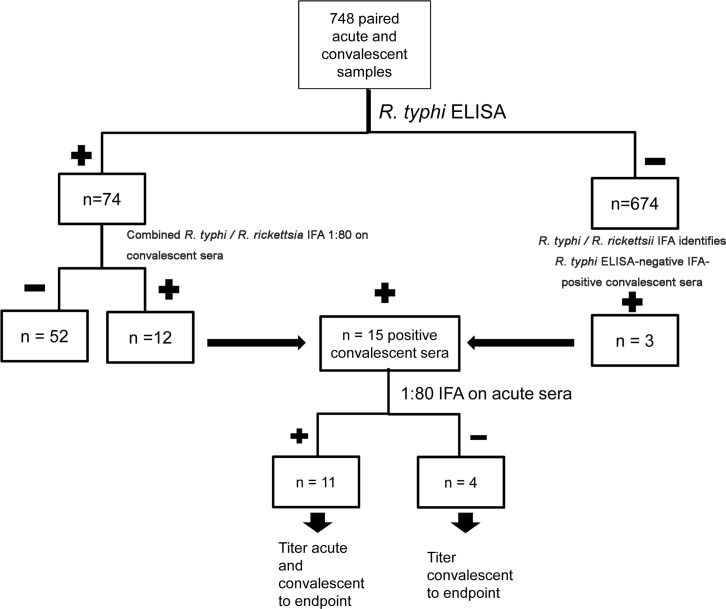
Flow diagram depicting approach to serological evaluation of convalescent and acute phase sera for IgG antibodies to *R*. *typhi*, including ELISA and IFA. Note that the IFA substrate slides used resulted in simultaneous tests for both *R*. *rickettsii* and *R*. *typhi*; those samples identified with *R*. *rickettsii* IFA antibodies were then included in the *R*. *rickettsii* tally (Fig 1) if not *R*. *rickettsii* ELISA reactive.

Testing convalescent sera by IgG ELISA for antibodies to *R*. *rickettsii* and *R*. *typhi* yielded 125 cases of possible rickettsioses (66 *R*. *typhi* ELISA-positive only, 51 *R*. *rickettsii* ELISA-positive only, and 8 positive for both). Acute and past rickettsioses identified by SFGR vs. TGR ELISA vs. both are shown in Figs 1 and 2. Of note, since the IFA substrate slides we used for *R*. *rickettsii* and *R*. *typhi* result in simultaneous tests for both, all positive by either ELISA were tested for both *R*. *rickettsii* and *R*. *typhi* by IFA. Of convalescent sera positive with the *R*. *typhi* ELISA but not the *R*. *rickettsii* ELISA, 15 had *R*. *rickettsii* IFA antibody titers ≥80 and 7 had *R*. *rickettsii* titers ≥160. Testing paired sera by IFA for both *R*. *rickettsii* and *R*. *typhi* identified 2 acute SFGR infections that would have gone undetected (one seroconversion with convalescent titer 160 and one 4-fold rise in titer from 160 to 1280).

#### Rickettsial infections detected by IFA and PCR

IFA testing of paired sera identified 7 confirmed acute rickettsial infections, including 6 acute SFGR and 1 acute TGR infections. Additionally, we identified 27 confirmed past rickettsial infections, including 25 SFGR, 1 TGR, and 1 SFGR/TGR. Finally, we identified 10 probable acute rickettsial infections (8 acute SFGR and 2 acute SFGR/TGR) and 8 probable past rickettsial infections (5 past SFGR and 3 past SFGR/TGR). Among those with acute SFGR infection, the IFA IgG geometric mean titer (GMT) increased 5-fold between the acute and convalescent samples, and the GMT for the convalescent samples was 320. One patient had an 8-fold increase in IFA IgG titer for *R*. *parkeri* (<40 acute, 320 convalescent) and stable acute and convalescent titers for *R*. *rickettsii* (320). The patient with an acute TGR infection had an IFA IgG acute titer that increased 4-fold (160 acute and 640 convalescent). A total of 33 of 49 DNA samples from acute-phase blood from patients with serologic reactivity potentially suggestive of SFR or TGR infection (seroconversion, a ≥4-fold rise in titer, or high (1:160) convalescent and/or acute phase titers) were tested by multiplex PCR and all were found to lack DNA evidence of SFGR or TGR rickettsemia. Eleven acute phase samples from patients with paired serology-confirmed acute SFGR or TGR infection or acute or convalescent titers ≥ 160 or seroconversion with 2 fold rise in titer were retested in a separate laboratory and were also found to lack DNA evidence of SFG or TG rickettsemia; all 14 of those with IFA results similarly suggestive of *C*. *burnetii* infection were also negative by PCR.

### Clinical features and epidemiology of rickettsial infections

#### Clinical features of acute rickettsial infection (Table 1)

Patients with laboratory-confirmed acute rickettsial infection were older (median age 55 vs. 9 years, p = 0.01) and reported a longer duration of fever than others with febrile illness (median 8 days vs. 2 days, p = 0.02). The only symptoms that differentiated acute rickettsial infections from other febrile illnesses were muscle and joint pains. Most patients who reported muscle and/or joint pain reported both, especially those with rickettsial infections (83.3% [5/6] of those with rickettsioses vs. 76.2% [192/252] of those without). In contrast, neither signs nor laboratory findings distinguished acute rickettsial infections from other acute febrile illness. No patient was suspected to have acute rickettsial infection; instead, 4 were suspected to have a focal bacterial infection, 2 an unclear febrile syndrome, and 1 a viral infection. Although two received an antibiotic (1 cephalosporin and 1 penicillin), none were treated with doxycycline. No patient with acute rickettsial infection died.

**Table 1:** Clinical characteristics of febrile patients with confirmed acute rickettsial infection vs. no acute rickettsial infection, Nicaragua.

Clinical Characteristics	Acute rickettsial infection (n = 7)	No acute rickettsial infection (n = 741)	P value*
Median age, years, (IQR)	55 (15,73)	9 (3,28)	0.01
Male sex	57%	53%	0.81
Male adult (≥ 15 years)	58%	47%	0.46
Days ill, median (IQR)	8.0 (2.0, 14.0)	3.0 (1.0, 5.0)	0.08
Days fever, median (IQR)	8.0 (2.0, 30.0)	2.0 (1.0, 4.0)	0.02
Prior antibiotic treatment	29%	30%	0.94
Admitted to hospital	71%	41%	0.10
**Symptoms**			
Headache	57%	50%	0.72
Chills	71%	63%	0.64
Sore throat	29%	29%	0.96
Cough	43%	39%	0.85
Dyspnea	14%	12%	0.88
Lethargy	29%	29%	0.21
Joint pain	86%	29%	0.001
Muscle pain	71%	32%	0.025
Abdominal pain	14%	27%	0.46
Vomiting	14%	36%	0.23
Diarrhea	0%	17%	0.23
Dysuria	29%	13%	0.23
Oliguria	14%	8%	0.53
**Signs**			
Conjunctival injection	0%	1%	0.83
Pharyngeal exudate	43%	29%	0.44
Lymphadenopathy	0%	21%	0.17
Jaundice	0%	1%	0.80
Lung crackles	14%	11%	0.80
Tender spleen	0%	1%	0.81
Tender liver	0%	3%	0.66
Hepatomegaly	0%	2%	0.69
Splenomegaly	0%	1%	0.83
Rash	0%	8%	0.44
Petechiae	0%	2%	0.71
Eschar	0%	1%	0.78
**Laboratory parameter**	Median (IQR)		
WBC per μl	10200 (6850–13800)	11700 (8500–16000)	0.25
ANC per μl	6936 (1987–11040)	8500 (5694, 12096)	0.15
ALC per μl	3264 (1768, 4864)	2327 (1500, 3624)	0.19
Platelets x 1000 per μl^	243 (195, 350)	272 (216, 346)	0.67

WBC, White blood count; ANC, Absolute neutrophil count; ALC, Absolute lymphocyte count; IQR, interquartile range; SD, standard deviation

*Kruskal-Wallis test for proportions and skewed continuous variables; analysis of variance test: normally distributed continuous variables. ^400 with data

#### Epidemiology of acute or past rickettsial infection (Table 2)

Overall, 34 (5%) of 748 patients had confirmed acute or past rickettsial infection. Acute rickettsial infections occurred disproportionately (p = 0.02) in cooler months during the dry season (6 confirmed in November and 1 in April). Those seropositive for rickettsioses were also more likely to report rural residence and less education. Similarly, those reporting exposure to farm animals (horses, cows, and pigs), domestic pets (dogs, cats, and birds), and/or ectoparasites (ticks, fleas, and lice) were also more likely to have acute or past rickettsial infection. Finally, reporting river exposure and drinking well water was associated with rickettsial infection.

**Table 2:** Epidemiologic characteristics of patients with definitive serologic evidence of rickettsial infection vs. no rickettsial infection, Nicaragua, 2008

Demographic Characteristics	Rickettsial infection (n = 34)	No rickettsial infection (n = 714)	P value*
Median age, years, (IQR)	51 (18,65)	9 (3,27)	<0.0001
Male sex	65%	52%	0.15
Male adult (≥15 years)	67%	46%	0.03
Rural Residence	53%	27%	0.001
**Education, if age** **≥15**			0.001
Illiterate	34%	11%	
Primary	41%	37%	
Secondary	21%	43%	
University	41%	21%	
Illiterate	3%	10%	
**Type of Work, if age** ≥15*			0.02
Home	70%	45%	
Student	3%	19%	
Worker	10%	17%	
Farmer	13%	5%	
Merchant	3%	7%	
Other	0%	7%	
**Animal exposures**			
Horse	41%	14%	<0.001
Cow	35%	12%	<0.001
Pig	47%	23%	0.001
Goat	6%	3%	0.37
Cat	53%	24%	<0.001
Dog	82%	64%	0.03
Rodent	74%	70%	0.70
Bird	62%	39%	0.007
Ticks	32%	11%	<0.001
Flea	15%	5%	0.03
Mosquito	88%	77%	0.12
Louse	29%	11%	0.001
**Swim/bathe/wade**			
River	29%	11%	0.001
Other fresh water	3%	1%	0.47
Water source*			0.003
Tap	53%	78%	
Well	47%	20%	
River	0%	1%	
Bottle/boiled	0%	1%	

*Does not sum to 100% secondary to rounding.

### Laboratory testing for Q fever

#### Serological screening for Q fever by ELISA

Testing of convalescent sera by Phase 2 IgG ELISA for *C*. *burnetii* yielded 31 cases of possible Q fever.

#### Q fever infections detected by IFA and PCR

IFA testing of paired sera from 30 of the 31 IgG ELISA-screen positive patients identified 10 confirmed acute Q fever infections, 4 confirmed past Q fever infections, and 1 probable past Q fever infection. Among those with acute Q fever infection, the IFA IgG geometric mean titer (GMT) increased 6.96 fold between the acute and convalescent samples, and the GMT for the convalescent samples was 485. DNA from acute-phase blood from 14 patients with rising titers to *C*. *burnetii* phase 2 antigen and a convalescent titer of ≥160 were tested by PCR and found to lack *C*. *burnetii* DNA.

### Clinical features and epidemiology of Q fever

#### Clinical features of Acute Q fever (Table 3)

Patients with acute Q fever were older than others with acute febrile illness (median 31 years vs. 9 years, respectively. p = 0.01), but a similar proportion were male (p = 0.63). Patients with acute Q fever reported longer durations of illness (median 7.5 vs 3.0 days, p = 0.01) and of reported fever (3.0 vs. 2.0 days, p = 0.04) than others with febrile illness. Patients with acute Q fever were more likely than others to have sore throat (60% vs. 29%, p = 0.03). Although cough was not more common in those with acute Q fever than others, patients were more likely to report a longer duration of cough (p = 0.04). Two (20%) with prolonged dry cough and physical findings on chest examination were found to have lobar pneumonia, one associated with pleural effusion; two others had negative chest radiographs, one of which had ascites. Patients with headache, dyspnea, and sore throat associated with acute Q fever tended to report longer durations as well (median 6 vs. 2 days, p = 0.06, median 15 vs 7 days, p = 0.05, and median 7 vs. 2.5 days, p = 0.09, respectively). Patients with acute Q fever were more likely than others to have hepatomegaly (20% vs. 2%, p<0.001) and splenomegaly (10% vs. 1%, p<0.001) but not jaundice. Leukopenia was not observed, but absolute lymphocyte count (ALC) tended to be lower (1617 vs. 2368 per μl, p = 0.05). No patient was clinically suspected to have acute Q fever. Eight of 10 were thought to have a focal bacterial infection, 1 bacteremia, and 1 leptospirosis. None were treated with doxycycline or with trimethoprim-sulfamethoxazole. No patient with acute Q fever died.

**Table 3:** Clinical characteristics of febrile patients with acute Q fever vs. no acute Q fever, Nicaragua.

Clinical Characteristics	Acute Q fever (n = 10)	No acute Q fever (n = 738)	P value*
Median age, years, (IQR)	31 (19,39)	9 (3,28)	0.01
Male sex	60%	52%	0.63
Male adult (≥15 years)	56%	48%	0.64
Days ill, median (IQR)	7.5 (3.0, 15.0)	3.0 (1.0, 5.0)	0.01
Days fever, median (IQR)	3.0 (2.0, 10.0)	2.0 (1.0, 4.0)	0.04
Prior antibiotic treatment	50%	30%	0.16
Admitted to hospital	33%	41%	0.64
Symptoms			
Headache	60%	50%	0.54
Chills	80%	63%	0.26
Sore throat	60%	29%	0.03
Cough	50%	39%	0.49
Days, median (IQR)	11.0 (5, 17.5)	3.0 (2.0, 6.0)	0.04
Dyspnea	30%	12%	0.09
Lethargy	0%	13%	0.22
Joint pain	40%	29%	0.46
Muscle pain	40%	32%	0.58
Abdominal pain	20%	27%	0.64
Vomiting	20%	36%	0.29
Diarrhea	0%	17%	0.15
Dysuria	10%	13%	0.76
Oliguria	0%	8%	0.35
Signs			
Conjunctival injection	0%	1%	0.79
Pharyngeal exudate	50%	29%	0.15
Lymphadenopathy	20%	21%	0.95
Jaundice	0%	1%	0.76
Lung crackles	20%	11%	0.38
Tender spleen	0%	1%	0.78
Tender liver	0%	3%	0.60
Hepatomegaly	20%	2%	<0.001
Splenomegaly	10%	1%	<0.001
Rash	0%	8%	0.35
Petechiae	0%	2%	0.66
Eschar	0%	1%	0.74
Laboratory parameter	Median (IQR)		
WBC per μl	10400 (7800–11000)	11750 (8500–16000)	0.43
ANC per μl	7332 (6310–8470)	8509 (5626, 12096)	0.83
ALC per μl	1617 (1320, 2100)	2368 (1508, 3654)	0.05
Platelets x 1000 per μl^	264 (199, 300)	272 (216, 347)	0.78

WBC, White blood count; ANC, Absolute neutrophil count; ALC, Absolute lymphocyte count; IQR, interquartile range; SD, standard deviation

*Kruskal-Wallis test for proportions and skewed continuous variables; analysis of variance test: normally distributed continuous variables. ^ 400 with data

### Epidemiology of acute or past Q fever (Table 4)

Overall, 14 (2%) of 747 patients had evidence of Q fever infection. Patients with definitive serologic evidence of Q fever infection were older than those without (median 36 vs. 9 years, p = 0.0003); 3.9% of adults age 18 years or older were seropositive for Q fever compared with 0.7% of children (p = 0.002). Acute Q fever occurred throughout the study period (1 each in October to March except for 3 in April and 2 in November) without seasonality or specific association with rainfall or temperature. Otherwise, despite extensive investigation, no demographic characteristics or environmental exposures were associated with Q fever. However, patients with Q fever infection were more likely than others to have definitive serologic evidence of rickettsial infection (21% vs 4%, p = 0.002).

**Table 4:** Epidemiologic characteristics of patients with definitive serologic evidence of Q fever vs. no Q fever, Nicaragua

Demographic Characteristics	Q fever (n = 14)	No Q fever (n = 734)	P value*
Median age, years, (IQR)	36 (19,59)	9 (3,28)	0.0003
Male sex	64%	52%	0.37
Male adult (≥15 years)	58%	47%	0.460
Residence			
Rural	36%	28%	0.51
Education, if age ≥15			0.67
Illiterate	25%	13%	
Primary	33%	37%	
Secondary	33%	41%	
University	8%	9%	
Type of Work, if age ≥15*			0.53
Home	67%	46%	
Student	17%	17%	
Worker	0%	17%	
Farmer	8%	6%	
Merchant	8%	6%	
Other	0%	7%	
Animal exposures			
Horse	14%	16%	0.90
Cow	14%	13%	0.88
Pig	36%	23%	0.28
Goat	7%	3%	0.40
Cat	29%	25%	0.75
Dog	79%	54%	0.28
Rodent	57%	71%	0.27
Bird	36%	40%	0.76
Ticks	7%	12%	0.58
Flea	0%	6%	0.35
Mosquito	86%	77%	0.44
Louse	14%	12%	0.75
Swim/bathe/wade			
River	21%	11%	0.23
Other fresh water	0%	2%	0.64
Water source			0.89
Tap	71%	77%	
Well	29%	21%	
River	0%	1%	
Bottle/boiled	0%	1%	

*Does not sum to 100% secondary to rounding.

## Discussion

Using stringent serodiagnostic criteria and achieving 90% follow-up, we document and describe *Rickettsia* spp. and *C*. *burnetii* as causes of acute febrile illness in Nicaragua. We required a 4-fold change in IgG titer to define a laboratory-confirmed case of acute rickettsial infection, which is in keeping with the latest (2010) case definition for acute spotted fever infection from the Centers for Disease and Prevention [6]. We found that SFGR and *C*. *burnetii* caused undifferentiated febrile illness predominantly in adults, especially male adults for SFGR. These infections mimicked other acute febrile illness and were both unsuspected and untreated, as we found in a similar cohort study in Sri Lanka [11]. Improved awareness and diagnostic tests may decrease morbidity and mortality by enhancing case detection and prompt provision of appropriate therapy.

It is plausible that rickettsioses, both SFGR and TGR, would cause acute febrile illness in Nicaragua. *R*. *rickettsii*, the cause of Rocky Mountain spotted fever (RMSF) is distributed broadly throughout the Western Hemisphere, and confirmed cases have been documented elsewhere in Central America (specifically Mexico, Costa Rica, and Panama) [12–18]. Although not found in this study, fatal infections with *R*. *rickettsii* have been reported in Mexico, Costa Rica and Panama [15–17,19], and fatal SFGR, presumably *R*. *rickettsii*, in Guatemala [14]. In Mexico, RMSF is directly linked to *R*. *rickettsii*-infected *Rhipicephalus sanguineus* ticks harbored by the large population of peridomestic stray dogs [20]; notably, in the U.S., this same vector-host dynamic has been associated with 4-times higher RMSF case-fatality among American Indians compared with other ethnic groups [21–24]. Moreover, other important vectors of *R*. *rickettsii* in Central and South America (*Amblyomma mixtum* and *A*. *sculptum*, respectively) are present in Nicaragua, which heightens the likelihood that the serologic responses we observed are the result of *R*. *rickettsii* infection [25]. *R*. *parkeri*, another SFGR, is not yet implicated in human SFGR infection in Central America, but *A*. *maculatum* is present in the region and *R*. *parkeri*-like illness was reported in a traveler returning from Honduras [25,26]. In Brazil, *Amblyomma ovale* is the vector of *Rickettsia sp*. strain Atlantic rainforest, a *R*. *parkeri*-like agent [19]. Although serologic cross-reactions occur among species of SFG rickettsiae and serologic testing alone cannot directly identify a causative agent, higher titers in most cases to *R*. *rickettsii* than to *R*. *parkeri* suggest that *R*. *rickettsii* or a novel agent more closely related to *R*. *rickettsii* may have caused the confirmed SFG rickettsial infections. The relatively mild clinical illness we observed suggests the latter.

Although *C*. *burnetii* is technically not a member of the Rickettsiales, the disease it causes, namely Q fever, is plausible as a cause of acute febrile illness in Nicaragua and is found worldwide when sought. Q fever was first identified in Central America (Panama) in the 1940s [27,28] and more recently a serosurvey of Q fever in livestock workers confirmed a seroprevalence as high as 10% [29]. A 1971 serosurvey of rickettsioses and Q fever in humans in Central America identified SFGR and Q fever antibodies by complement fixation and microagglutination and found an SFGR seroprevalence of 0.3% (1/312) and Q fever seroprevalence of 0.6%-1.0% [2].

We found that patients with acute rickettsial infections had illnesses and findings that closely resembled those of other patients with acute febrile illness. Patients with rickettsioses, however, were relatively older, reported a longer duration of fever, and more frequently had joint and muscle pain than did others; no patient had a rash. Although fever, headache, and rash constitute the classic clinical triad for rickettsioses, headache is frequent with other illnesses and rash is often absent when patients present early in illness, as in our cohort, or are individuals with dark skin [30]. Our group and others have also found that clinical characteristics are often not helpful in identifying rickettsial infections, with the exception possibly of older age [11,31–35]. In our study, acute rickettsial infections (almost all SFGR) were associated with self-reported rural residence, contact with livestock, and drinking well or river versus tap water. Tick-borne rickettsial infections are increasingly recognized as zoonotic infections that are more common in rural areas with complex tick-animal reservoir relationships that merit One Health approaches for control [11,36,37]. Patients with rickettsioses were also more likely to report exposure to ticks, fleas, and lice but not mosquitos. These findings are also plausible, since ticks and fleas harbor SFGR, and fleas and lice TGR. Patients with Q fever were also relatively older than other patients, but perhaps surprisingly did not report rural residence or exposure to livestock. We suspect this reflects small sample size, since individuals with confirmed acute Q fever did have acute Q fever-compatible illnesses, including hepatomegaly and prolonged illness associated with fever and cough.

We found that both acute rickettsial infections and Q fever were unsuspected and untreated, despite the availability of doxycycline and its use in a few patients without rickettsioses. In addition to non-specific findings, rickettsioses and Q fever pose a diagnostic and therapeutic challenge because paired serology, the reference standard, is intrinsically retrospective and current PCR protocols are insensitive. Our ability to examine and identify distinguishing clinical features if present was greatly improved by the >90% follow-up albeit limited by sample size. Although many SFGR infections and Q fever cases are self-limited, unconfirmed and potentially fatal rickettsial infections could have occurred in the 10% lost to follow-up, the unknown number of individuals too sick or poor to reach the hospital, and among the 5 who reported doxycycline treatment (since treatment can dampen the IgG immune response) [38]. Severe disease and deaths due to confirmed and presumed RMSF have been reported in Central America [15–18], [12–16] with case fatality up to 20% [39]. However, the diversity, relative frequency, and clinical spectrum of SFG rickettsioses is not known in Central America, nor are the determinants of disease severity among SFG rickettsioses

Conservatively, 5% (34/748) of patients with AFI had definitive evidence of rickettsial infection and 2% (14/748) Q fever. Our estimate is conservative because our algorithm of confirming ELISA positives with reference standard IFA assured specificity but not sensitivity, the latter of which would have required performing IFA in all patients. We required an IFA titer of 160 to be positive because we preferred to underreport rather than falsely report *Rickettsia* spp. and *C*. *burnetii* as newly identified causes of acute febrile illness in Nicaragua. However, it is likely that these etiologic agents caused much more than 6% of acute febrile illnesses and that the seroprevalence of rickettsioses and Q fever together was at least 10%. Because IFA is inherently subjective and readings can vary by one dilution even among experts [30],we conservatively required a convalescent titer of 160 to define confirmed infection as we have done previously [11], including in the setting of seroconversion. Confirmed acute rickettsial infections required demonstration of a 4-fold rise in IgG by IFA on paired sera, which is in consistent with the US Centers for Disease Control and Prevention’s decision to no longer accept a single high IFA titer or a positive ELISA as confirmatory for *R*. *rickettsii* infection [34]. We ascribed infections to SFGR rather than TGR if the former had 2-fold higher titers as we have done previously [11]. Although cross-reactions occur between groups and especially species within groups, titers would be expected to be higher in the homologous group. In addition to our stringent case definitions, it is likely that cases were missed because of lack of access to care and possibly also due to early treatment or mortality.

A major strength of our study is use of SFG and TG ELISA to identify probable rickettsial infections followed by SFG and TG IFA on paired specimens to confirm acute vs. past infections. In contrast with existing seroepidemiological studies of rickettsial infections that solely utilize ELISA performed on a single serum sample, we opted to use a two-tiered approach that leverages the strengths of ELISA methods (high throughput and objective evaluation) but retains IFA (gold standard, but somewhat cumbersome and subjective) to strengthen certainty of our data. Our approach is in keeping with the US Centers for Disease Control and Prevention’s latest case definition [6] that classifies ELISA-positive cases as probable, and requires a 4-fold rise in titer by IFA to confirm SFG infections. We additionally found that ELISA screening should include both SFG and TG antigen, since two of 6 patients diagnosed with acute spotted fever rickettsiosis would have been missed if simultaneous IFA screening for both typhus and spotted fever group rickettsiae was not conducted. It is possible that we missed additional acute rickettsial infections that would have been identified had we performed IFA on the full cohort; hence, because of ELISA’s imperfect sensitivity in addition to specificity, we would emphasize the importance of IFA and empiric treatment for management of patients with suspected rickettsial infections. Given the limitations of serology, we also sought to confirm cases by PCR; however, PCR on whole blood is intrinsically insensitive for rickettsioses, due to low levels of bacteremia and the intracellular location of these bacteria within endothelial cells [40,41]. PCR for *Coxiella* is also insensitive, especially among seropositive patients [42,43]. Therefore, it is not surprising that paired IFA-confirmed infections were not corroborated by PCR.

In summary, we provide definitive evidence and a conservative estimate of unsuspected and untreated rickettsial infections and Q fever among patients with AFI in Nicaragua. A population-based longitudinal study with speciation of SFGR will be required to define the full clinical spectrum of *R*. *rickettsii* vs. other possible SFGR species and the case fatality rate of specific SFGR, TGR, and Q fever in Nicaragua. Better diagnostic tests, evaluated relative to gold standard paired serology such as we achieved here, and further epidemiologic study will be necessary to understand the biology of human rickettsioses and Q fever in Central America, to identify vector-host relationships, and to guide treatment and preventive measures.

## Supporting Information

S1 Checklist (DOC)
